# Supplementary material for: Measurement invariance of the strengths and difficulties questionnaire across socioeconomic status and ethnicity from ages 3 to 17 years: A population cohort study
Source: PLoS One. 2022 Dec 30;17(12):e0278385. doi: 10.1371/journal.pone.0278385 (PMC9803244; doi:10.1371/journal.pone.0278385)
Supplement: S1 File — (DOCX) [file pone.0278385.s001.docx]

**Supporting Information**

**Strengths and difficulties questionnaires items**

**Emotional problems**

The items were: ***“****often complains of headaches, stomach aches, or sickness”*, *“has many worries, often seems worried”*, *“often unhappy, downhearted, or tearful”*, *“nervous or clingy in new situations, easily loses confidence”*, *“has many fears, easily scared”*.

**Peer problems**

The items were: *“rather solitary, tends to play alone”*, *“has at least one good friend”*, *“generally liked by other children”*, *“picked on or bullied by other children”*, *“gets on better with adults than other children”*.

**Conduct problems**

The items were: *“often has temper tantrums or hot tempers”,* *“usually obedient, does what adults request”*, *“often fights with other children or bullies them”*, *“often lies or cheats”* (at age three this was replaced with *“argumentative with adults”*), *“steals from home, school, or elsewhere”* (at age three this was replaced with *“can be spiteful”*).

**Hyperactivity/inattention**

The items were: *“restless, overactive, cannot stay still for long”*, *“constantly fidgeting or squirming”*, *“easily distracted, concentration wanders”*, *“thinks things out before acting”* (at age three this was replaced with *“can stop and think before acting”*), *“sees tasks through to the end, good attention span”*.

**Prosocial behaviour**

The items were: *“considerate of other people’s feelings”*, *“shares readily with other children”*, *“helpful if someone is hurt, upset, or feeling ill”*, *“kind to younger children”*, *“often volunteers to help others”*.

**Table S1. Descriptive Statistics for the Emotional Problems Subscale**

|  | **Age 3** | **Age 5** | **Age 7** | **Age 11** | **Age 14** | **Age 17** |
| --- | --- | --- | --- | --- | --- | --- |
| **Overall** | 1.334 (1.438) | 1.365  (1.556) | 1.509 (1.735) | 1.852 (1.977) | 2.027 (2.119) | 2.023 (2.222) |
| **Household Income** |  |  |  |  |  |  |
| Lowest Quintile | 1.762 (1.684) | 1.741 (1.759) | 1.934 (1.973) | 2.220 (2.141) | 2.589 (2.317) | 2.525 (2.378) |
| 2nd Quintile | 1.505 (1.574) | 1.476 (1.624) | 1.646 (1.820) | 2.076 (2.059) | 2.241 (2.144) | 2.260 (2.257) |
| 3rd Quintile | 1.211 (1.324) | 1.255 (1.467) | 1.437 (1.667) | 1.801 (1.919) | 1.947 (2.095) | 1.951 (2.196) |
| 4th Quintile | 1.117 (1.229) | 1.169 (1.415) | 1.298 (1.539) | 1.654 (1.881) | 1.758 (1.944) | 1.807 (2.122) |
| Highest Quintile | 1.016 (1.120) | 1.081 (1.314) | 1.145 (1.450) | 1.424 (1.710) | 1.524 (1.862) | 1.566 (2.004) |
| **Parental Highest Education** | |  |  |  |  |  |
| O Level/ GCSE Grade D-G | 1.534 (1.520) | 1.595 (1.654) | 1.775 (1.959) | 2.100 (2.081) | 2.380 (2.251) | 2.426 (2.382) |
| O Level/ GCSE Grade A*-C | 1.283 (1.354) | 1.271 (1.448) | 1.456 (1.697) | 1.912 (2.020) | 2.022 (2.148) | 2.072 (2.276) |
| A / AS Levels & Equivalent | 1.123 (1.228) | 1.151 (1.403) | 1.274 (1.578) | 1.665 (1.921) | 1.795 (1.957) | 1.878 (2.147) |
| Diploma in Higher Education & Equivalent | 1.056 (1.181) | 1.162 (1.441) | 1.366 (1.570) | 1.625 (1.815) | 1.804 (2.011) | 1.774 (2.144) |
| First degree or Higher | 1.036 (1.179) | 1.137 (1.377) | 1.198 (1.454) | 1.448 (1.716) | 1.548 (1.840) | 1.599 (1.983) |
| **Ethnicity** |  |  |  |  |  |  |
| White | 1.272 (1.368) | 1.311 (1.511) | 1.470 (1.706) | 1.852 (1.988) | 2.002 (2.138) | 2.023 (2.252) |
| Mixed | 1.377 (1.494) | 1.523 (1.597) | 1.608 (1.839) | 1.965 (2.083) | 2.079 (2.059) | 2.172 (2.411) |
| South Asian | 1.966 (1.888) | 1.803 (1.860) | 1.856 (1.903) | 1.936 (1.882) | 2.222 (1.991) | 2.011 (1.990) |
| Black | 1.354 (1.558) | 1.392 (1.524) | 1.416 (1.710) | 1.477 (1.791) | 1.731 (1.896) | 1.800 (1.906) |
| Other | 1.738 (1.719) | 1.875 (1.920) | 1.910 (1.981) | 1.874 (1.937) | 2.508 (2.244) | 2.211 (2.215) |

Values are mean (standard deviation)

**Table S2. Descriptive Statistics for the Peer Problems Subscale**

|  | **Age 3** | **Age 5** | **Age 7** | **Age 11** | **Age 14** | **Age 17** |
| --- | --- | --- | --- | --- | --- | --- |
| **Overall** | 1.453 (1.465) | 1.124 (1.374) | 1.187 (1.482) | 1.353 (1.659) | 1.723 (1.794) | 1.754  (1.776) |
| **Household Income** | |  |  |  |  |  |
| Lowest Quintile | 1.870 (1.594) | 1.553 (1.526) | 1.662 (1.658) | 1.823 (1.793) | 2.243 (1.891) | 2.246 (1.867) |
| 2nd Quintile | 1.660 (1.532) | 1.296 (1.431) | 1.374 (1.551) | 1.596 (1.751) | 1.963 (1.815) | 1.982 (1.755) |
| 3rd Quintile | 1.430 (1.421) | 1.021 (1.311) | 1.101 (1.392) | 1.277 (1.593) | 1.642 (1.770) | 1.701 (1.756) |
| 4th Quintile | 1.200 (1.313) | .883 (1.204) | .927 (1.327) | 1.059 (1.503) | 1.437 (1.685) | 1.516 (1.711) |
| Highest Quintile | 1.034 (1.245) | .735 (1.138) | .773 (1.216) | .915 (1.403) | 1.248 (1.598) | 1.329 (1.637) |
| **Parental Highest Education** | |  |  |  |  |  |
| O Level/ GCSE Grade D-G | 1.667 (1.457) | 1.368 (1.466) | 1.398 (1.564) | 1.596 (1.739) | 2.075 (1.996) | 2.175 (1.897) |
| O Level/ GCSE Grade A*-C | 1.429 (1.416) | 1.048 (1.315) | 1.164 (1.449) | 1.335 (1.665) | 1.736 (1.807) | 1.821 (1.789) |
| A / AS Levels & Equivalent | 1.225 (1.371) | .927 (1.232) | 1.004 (1.387) | 1.170 (1.606) | 1.521 (1.708) | 1.635 (1.753) |
| Diploma in Higher Education & Equivalen | 1.225 (1.332) | .900 (1.219) | .906 (1.294) | 1.116 (1.539) | 1.487 (1.686) | 1.473 (1.650) |
| First degree or higher | 1.080 (1.322) | .788 (1.181) | .806 (1.266) | .966 (1.460) | 1.255 (1.595) | 1.344 (1.655) |
| **Ethnicity** | |  |  |  |  |  |
| White | 1.372 (1.414) | 1.046 (1.333) | 1.105 (1.451) | 1.308 (1.673) | 1.683 (1.828) | 1.725 (1.812) |
| Mixed | 1.530 (1.504) | 1.269 (1.389) | 1.445 (1.644) | 1.535 (1.790) | 1.871 (1.819) | 1.853 (1.751) |
| South Asian | 2.196 (1.708) | 1.722 (1.536) | 1.798 (1.551) | 1.632 (1.491) | 1.973 (1.557) | 1.940 (1.535) |
| Black | 1.653 (1.455) | 1.439 (1.497) | 1.400 (1.456) | 1.399 (1.463) | 1.681 (1.538) | 1.763 (1.593) |
| Other | 2.058 (1.785) | 1.641 (1.566) | 1.693 (1.625) | 1.811 (1.667) | 1.975 (1.707) | 1.855 (1.642) |

Values are mean (standard deviation)

**Table S3. Descriptive Statistics for the Conduct Problems Subscale**

|  | **Age 3** | **Age 5** | **Age 7** | **Age 11** | **Age 14** | **Age 17** |
| --- | --- | --- | --- | --- | --- | --- |
| **Overall** | 2.743 (2.009) | 1.491 (1.476) | 1.372 (1.514) | 1.372 (1.549) | 1.404 (1.615) | 1.167  (1.471) |
| **Household Income** | |  |  |  |  |  |
| Lowest Quintile | 3.453 (2.221) | 2.014 (1.697) | 1.893 (1.724) | 1.886 (1.774) | 1.980 (1.889) | 1.594 (1.691) |
| 2nd Quintile | 3.064 (2.106) | 1.670 (1.516) | 1.565 (1.602) | 1.637 (1.688) | 1.597 (1.667) | 1.343 (1.542) |
| 3rd Quintile | 2.622 (1.895) | 1.386 (1.399) | 1.277 (1.442) | 1.241 (1.430) | 1.280 (1.531) | 1.110 (1.432) |
| 4th Quintile | 2.364 (1.730) | 1.185 (1.232) | 1.106 (1.292) | 1.097 (1.333) | 1.137 (1.415) | .938 (1.331) |
| Highest Quintile | 2.103 (1.702) | 1.047 (1.183) | .923 (1.182) | .893 (1.152) | .952 (1.239) | .848 (1.182) |
| **Parental Highest Education** | |  |  |  |  |  |
| O Level/ GCSE Grade D-G | 3.192 (2.079) | 1.830 (1.555) | 1.758 (1.654) | 1.755 (1.694) | 1.888 (1.865) | 1.539 (1.746) |
| O Level/ GCSE Grade A*-C | 2.756 (1.926) | 1.470 (1.418) | 1.362 (1.494) | 1.391 (1.534) | 1.412 (1.631) | 1.180 (1.483) |
| A / AS Levels & Equivalent | 2.369 (1.749) | 1.221 (1.295) | 1.107 (1.320) | 1.121 (1.332) | 1.102 (1.414) | .997 (1.325) |
| Diploma in Higher Education & Equivalent | 2.362 (1.756) | 1.211 (1.271) | 1.114 (1.265) | 1.146 (1.391) | 1.171 (1.380) | .968 (1.314) |
| First degree or higher | 2.080 (1.707) | 1.028 (1.199) | .898 (1.161) | .880 (1.157) | .916 (1.191) | .807 (1.185) |
| **Ethnicity** | |  |  |  |  |  |
| White | 2.744 (2.008) | 1.485 (1.479) | 1.370 (1.522) | 1.375 (1.561) | 1.385 (1.619) | 1.136 (1.475) |
| Mixed | 2.868 (2.014) | 1.647 (1.491) | 1.459 (1.533) | 1.535 (1.763) | 1.590 (1.915) | 1.212 (1.590) |
| South Asian | 2.851 (2.086) | 1.522 (1.467) | 1.425 (1.433) | 1.359 (1.412) | 1.525 (1.543) | 1.337 (1.378) |
| Black | 2.443 (1.810) | 1.403 (1.419) | 1.190 (1.450) | 1.201 (1.394) | 1.243 (1.399) | 1.167 (1.495) |
| Other | 2.403 (1.938) | 1.514 (1.431) | 1.392 (1.532) | 1.279 (1.444) | 1.553 (1.553) | 1.476 (1.455) |

Values are mean (standard deviation)

**Table S4. Descriptive Statistics for the Inattention/Hyperactivity Subscale**

|  | **Age 3** | **Age 5** | **Age 7** | **Age 11** | **Age 14** | **Age 17** |
| --- | --- | --- | --- | --- | --- | --- |
| **Overall** | 3.736 (2.289) | 3.234 (2.337) | 3.314 (2.487) | 3.091 (2.456) | 2.964 (2.390) | 2.472 (2.251) |
| **Household Income** | |  |  |  |  |  |
| Lowest Quintile | 4.352 (2.391) | 3.861 (2.449) | 4.005 (2.593) | 3.727 (2.584) | 3.683 (2.497) | 3.108 (2.399) |
| 2nd Quintile | 4.031 (2.346) | 3.515 (2.386) | 3.572 (2.499) | 3.421 (2.531) | 3.263 (2.411) | 2.737 (2.276) |
| 3rd Quintile | 3.670 (2.180) | 3.149 (2.286) | 3.278 (2.489) | 3.061 (2.412) | 2.895 (2.378) | 2.456 (2.220) |
| 4th Quintile | 3.415 (2.177) | 2.867 (2.177) | 2.944 (2.363) | 2.668 (2.296) | 2.577 (2.264) | 2.135 (2.133) |
| Highest Quintile | 3.110 (2.106) | 2.577 (2.090) | 2.646 (2.213) | 2.440 (2.162) | 2.297 (2.104) | 1.928 (2.004) |
| **Parental Highest Education** | |  |  |  |  |  |
| O Level/ GCSE Grade D-G | 4.246 (2.376) | 3.774 (2.413) | 3.852 (2.557) | 3.653 (2.535) | 3.620 (2.566) | 3.076 (2.418) |
| O Level/ GCSE Grade A*-C | 3.795 (2.212) | 3.304 (2.305) | 3.402 (2.467) | 3.251 (2.485) | 3.086 (2.393) | 2.554 (2.263) |
| A / AS Levels & Equivalent | 3.469 (2.151) | 2.999 (2.272) | 3.014 (2.414) | 2.857 (2.372) | 2.692 (2.235) | 2.247 (2.226) |
| Diploma in Higher Education & Equivalent | 3.459 (2.181) | 2.901 (2.164) | 3.060 (2.359) | 2.783 (2.298) | 2.671 (2.234) | 2.251 (2.108) |
| First degree or higher | 2.888 (2.088) | 2.365 (2.026) | 2.520 (2.233) | 2.258 (2.151) | 2.112 (2.102) | 1.826 (1.958) |
| **Ethnicity** |  |  |  |  |  |  |
| White | 3.707 (2.286) | 3.217 (2.348) | 3.310 (2.511) | 3.105 (2.483) | 2.953 (2.431) | 2.450 (2.275) |
| Mixed | 3.822 (2.335) | 3.511 (2.453) | 3.443 (2.473) | 3.262 (2.537) | 3.057 (2.415) | 2.491 (2.330) |
| South Asian | 4.133 (2.282) | 3.366 (2.222) | 3.445 (2.284) | 3.064 (2.248) | 3.092 (2.082) | 2.682 (2.078) |
| Black | 3.577 (2.185) | 3.163 (2.255) | 2.982 (2.318) | 2.781 (2.231) | 2.788 (2.198) | 2.326 (1.994) |
| Other | 3.583 (2.397) | 3.105 (2.144) | 3.238 (2.388) | 2.737 (2.257) | 2.853 (2.315) | 2.470 (2.212) |

Values are mean (standard deviation)

**Table S5**. **Descriptive Statistics for the Prosocial Behaviour Subscale**

|  | **Age 3** | **Age 5** | **Age 7** | **Age 11** | **Age 14** | **Age 17** |
| --- | --- | --- | --- | --- | --- | --- |
| **Overall** | 7.086 (2.056) | 8.296 (1.757) | 8.523 (1.707) | 8.744 (1.614) | 8.274 (1.866) | 8.347 (1.847) |
| **Household Income** | |  |  |  |  |  |
| Lowest Quintile | 6.880 (2.182) | 8.094 (1.898) | 8.230 (1.918) | 8.445 (1.832) | 7.921 (2.067) | 8.046 (2.048) |
| 2nd Quintile | 6.942 (2.139) | 8.162 (1.808) | 8.398 (1.771) | 8.627 (1.731) | 8.154 (1.933) | 8.239 (1.870) |
| 3rd Quintile | 7.074 (1.988) | 8.334 (1.740) | 8.643 (1.615) | 8.874 (1.480) | 8.374 (1.782) | 8.415 (1.773) |
| 4th Quintile | 7.253 (1.955) | 8.452 (1.653) | 8.694 (1.551) | 8.899 (1.471) | 8.455 (1.745) | 8.484 (1.745) |
| Highest Quintile | 7.325 (1.948) | 8.509 (1.583) | 8.698 (1.562) | 8.939 (1.396) | 8.513 (1.693) | 8.552 (1.730) |
| **Parental Highest Education** | |  |  |  |  |  |
| O Level/ GCSE Grade D-G | 6.851 (2.099) | 8.107 (1.819) | 8.406 (1.770) | 8.633 (1.680) | 8.056 (1.900) | 8.115 (1.966) |
| O Level/ GCSE Grade A*-C | 7.102 (1.980) | 8.357 (1.711) | 8.570 (1.678) | 8.792 (1.562) | 8.291 (1.835) | 8.305 (1.871) |
| A / AS Levels & Equivalent | 7.126 (2.007) | 8.403 (1.619) | 8.649 (1.560) | 8.878 (1.503) | 8.434 (1.786) | 8.435 (1.817) |
| Diploma in Higher Education & Equivalent | 7.328 (1.941) | 8.473 (1.617) | 8.732 (1.520) | 8.892 (1.451) | 8.456 (1.740) | 8.546 (1.700) |
| First degree or higher | 7.352 (1.982) | 8.485 (1.657) | 8.664 (1.597) | 8.902 (1.433) | 8.461 (1.737) | 8.541 (1.739) |
| **Ethnicity** |  |  |  |  |  |  |
| White | 7.097 (1.368) | 8.320 (1.725) | 8.549 (1.678) | 8.786 (1.566) | 8.296 (1.845) | 8.356 (1.849) |
| Mixed | 7.153 (2.102) | 8.398 (1.771) | 8.501 (1.753) | 8.596 (1.826) | 8.098 (1.942) | 8.194 (1.975) |
| South Asian | 6.845 (2.286) | 7.994 (1.939) | 8.210 (1.922) | 8.438 (1.880) | 8.167 (1.965) | 8.343 (1.761) |
| Black | 7.443 (2.185) | 8.388 (1.867) | 8.655 (1.689) | 8.777 (1.560) | 8.279 (1.929) | 8.293 (1.912) |
| Other | 6.864 (2.461) | 8.057 (2.172) | 8.571 (1.927) | 8.414 (2.014) | 8.137 (2.004) | 8.283 (1.861) |

Values are mean (standard deviation)

**Table S6.** **Cronbach’s Alpha and McDonald’s Omega**

|  | **Emotional Problems** | **Peer**  **Problems** | **Conduct Problems** | **Hyperactivity** | **Prosocial Behaviour** |
| --- | --- | --- | --- | --- | --- |
| **Cronbach’s Alpha** | |  |  |  |  |
| Age 3 years | .522 | .468 | .680 | .712 | .656 |
| Age 5 years | .594 | .515 | .559 | .762 | .673 |
| Age 7 years | .651 | .580 | .603 | .786 | .701 |
| Age 11 years | .713 | .641 | .626 | .791 | .673 |
| Age 14 years | .722 | .619 | .647 | .773 | .736 |
| Age 17 years | .757 | .606 | .613 | .765 | .728 |
| **McDonald’s Omega** | |  |  |  |  |
| Age 3 years | .557 | .474 | .691 | .719 | .660 |
| Age 5 years | .619 | .521 | .574 | .766 | .675 |
| Age 7 years | .667 | .589 | .629 | .790 | .705 |
| Age 11 years | .726 | .658 | .659 | .794 | .681 |
| Age 14 years | .735 | .639 | .682 | .776 | .746 |
| Age 17 years | .767 | .633 | .657 | .768 | .741 |
